# Dental Extrusion with Orthodontic Miniscrew Anchorage: A Case Report Describing a Modified Method

**DOI:** 10.1155/2015/909314

**Published:** 2015-02-02

**Authors:** Ricardo Fidos Horliana, Anna Carolina Ratto Tempestini Horliana, Alexandre do Vale Wuo, Flávio Eduardo Guillin Perez, Jorge Abrão

**Affiliations:** ^1^School of Dentistry, Santa Cecilia University, Santos, Brazil; ^2^Departamento da Saúde, Nove de Julho University (UNINOVE), R. Vergueiro 235/249, Liberdade 01504-001, São Paulo, SP, Brazil; ^3^Department of Stomatology, College of Dentistry, University of São Paulo, Brazil; ^4^Department of Orthodontics and Pediatric Dentistry, University of São Paulo, Brazil

## Abstract

In recent years, the skeletal anchorage through miniscrews has expanded the treatment options in orthodontics (Yamaguchi et al., 2012). We hereby present a modified method for tooth extrusion for cases where crown-lengthening surgery is contraindicated for aesthetic reasons. This modified method uses three orthodontic appliances: a mini-implant, an orthodontic wire, and a bracket. The aim of this case report was to increase the length of the clinical crown of a fractured tooth (tooth 23) by means of an orthodontic extrusion with the modified method of Roth and Diedrich.

## 1. Introduction

For many years, the removal of bone or gingival tissues has been the most common method used for crown-lengthening surgery [1, 2]. This surgical procedure usually causes an uneven contour of the gingival margin in the anterior region. In addition, as fear of pain is one of the major problems in dentistry, patients often reject this traumatic surgery [3]. In recent years, as an alternative to such a highly invasive technique, miniscrews have been used as temporary anchorage devices (TAD) for several orthodontic tooth movements including forced eruption [4–6]. A recent case report by Roth et al. [7] demonstrated the successful application of an orthodontic miniscrew implant as anchorage for the extrusion of a fixed prosthesis of 3 elements (two teeth and one edentulous area between them).

However, the specific mechanics for extrusion of only one tooth adjacent to an edentulous area has not been developed yet.

The aim of this case report was to increase the clinical crown of the fractured tooth (tooth 23) by means of orthodontic extrusion with the modified method of Roth and Diedrich. Once the biologic width was reestablished, the tooth was restored with an intraradicular retainer and a metal-ceramic crown.

## 2. Case Presentation

### 2.1. History and Diagnosis

**Figure 1:**
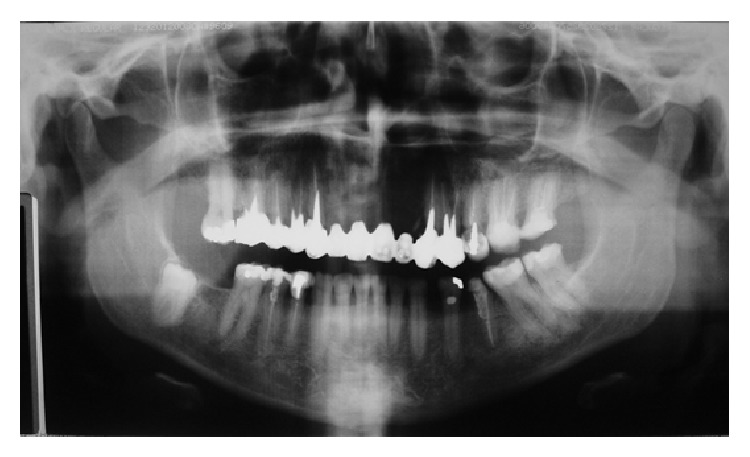
Findings of initial panoramic radiograph. Insufficient length of filling material of teeth 23, 24, 25, 26, 27, 15, 16, 45, 44, and 35. Old long fixed prosthesis included teeth 23, 24, 13, 14 15, and 16. Absent anterior teeth (22, 21, 11, and 12).

**Figure 2:**
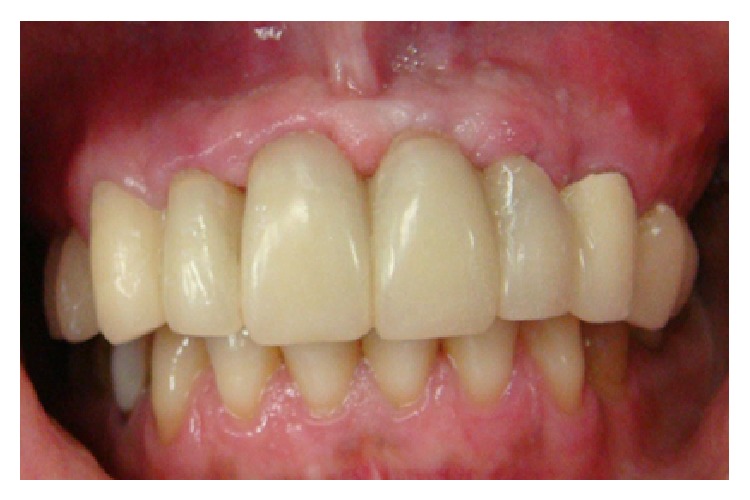
Clinical findings prior to treatment: inadequate prosthetic reconstruction in the anterior upper jaw.

**Figure 3:**
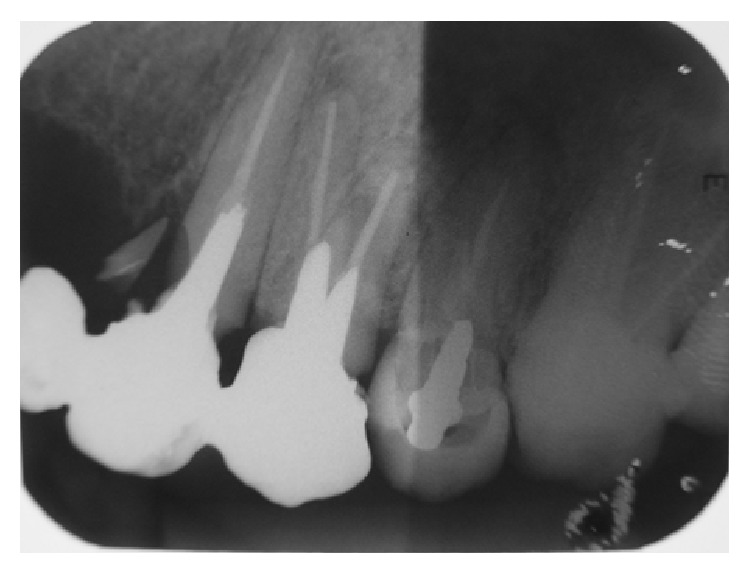
In this radiograph, there is a mesial fracture on the cervical third of tooth 23. Considering the long span of the fixed prosthesis, it could have overloaded the canine. In addition, insufficient length of the intraradicular retainer could have promoted inadequate tension distribution along the root. Also shown is the fixed partial denture supported by teeth 23 and 24. Tooth 25 presents a provisional prosthetic crown supported by a prefabricated pin. All teeth (23, 24, 25, and 26) show unsuccessful endodontic treatment.

A 51-year-old woman gave her informed consent for the case report to be published as advised by the University of São Paulo. The initial clinical findings demonstrated a prosthetic rehabilitation of a 26-year-old fixed partial denture from the right upper cuspid to the left upper cuspid (Figures 1 and 2) and a medial intraosseous fracture of the left upper cuspid (Figure 3).

**Figure 4:**
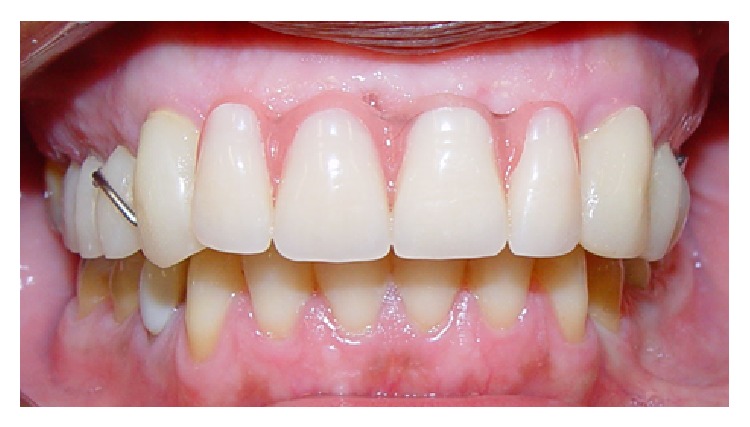
Removable partial temporary prosthesis installed after removing the fixed prosthesis.

**Figure 5:**
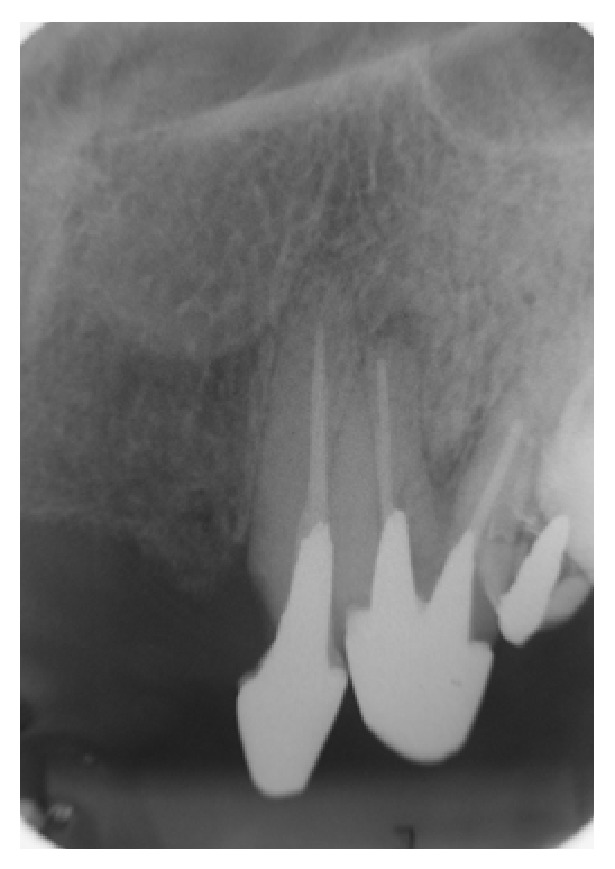
The medial fracture of tooth 23 was surgically extracted.

**Figure 6:**
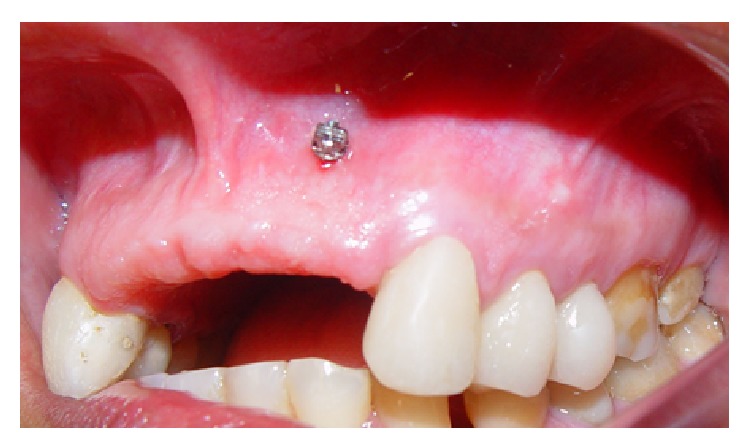
Bracket Top TAD miniscrew inserted vestibularly into the alveolar bone of the region of tooth 22.

After instructions on oral hygiene and plaque removal, the original prosthesis was replaced by a removable temporary partial prosthesis (Figure 4) and the medial fracture of the left upper cuspid was surgically extracted (Figure 5). Subsequently, under local anesthesia (mepivacaine 0.4 mL), a 2.0 mm diameter and 6.0 mm length miniscrew (Bracket Top TAD; Rocky Mountain Orthodontics, CO, USA) was inserted in the vestibular portion of the alveolar bone of the left upper lateral incisor. The miniscrew was safely inserted in the edentulous region on the medial cuspid side (Figure 6).

The periapical radiograph made prior to the treatment was used as a guide for the correct placement of the miniscrew. Normally, presurgical computerized tomography (CT) of surgical guides would be required to facilitate the safe placement of the miniscrew between the roots or when anatomic devices are present. However, CT was not needed in this specific case as we were dealing with an edentulous area [6].

**Figure 7:**
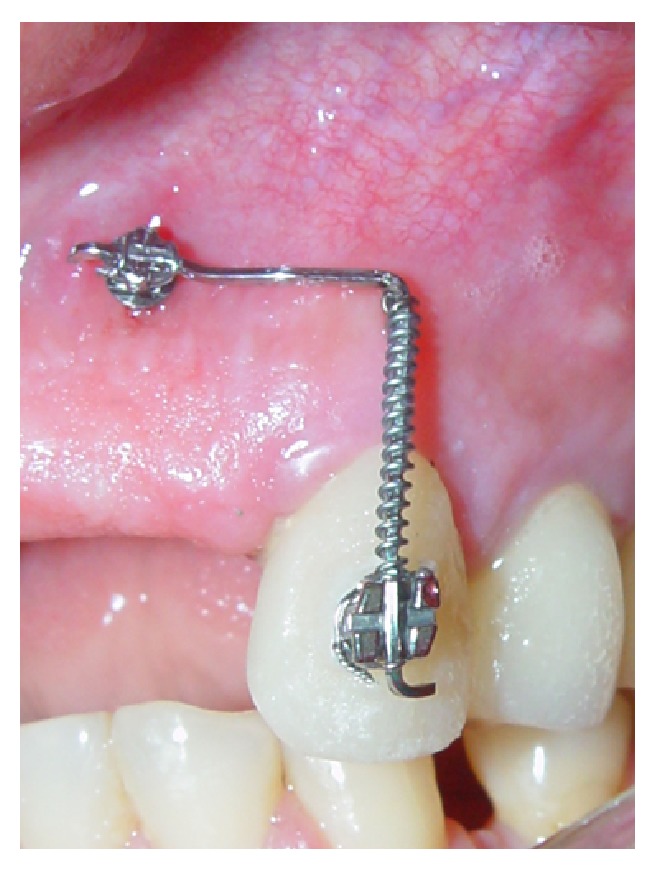
Clinical illustration of extrusion appliance.

**Figure 8:**
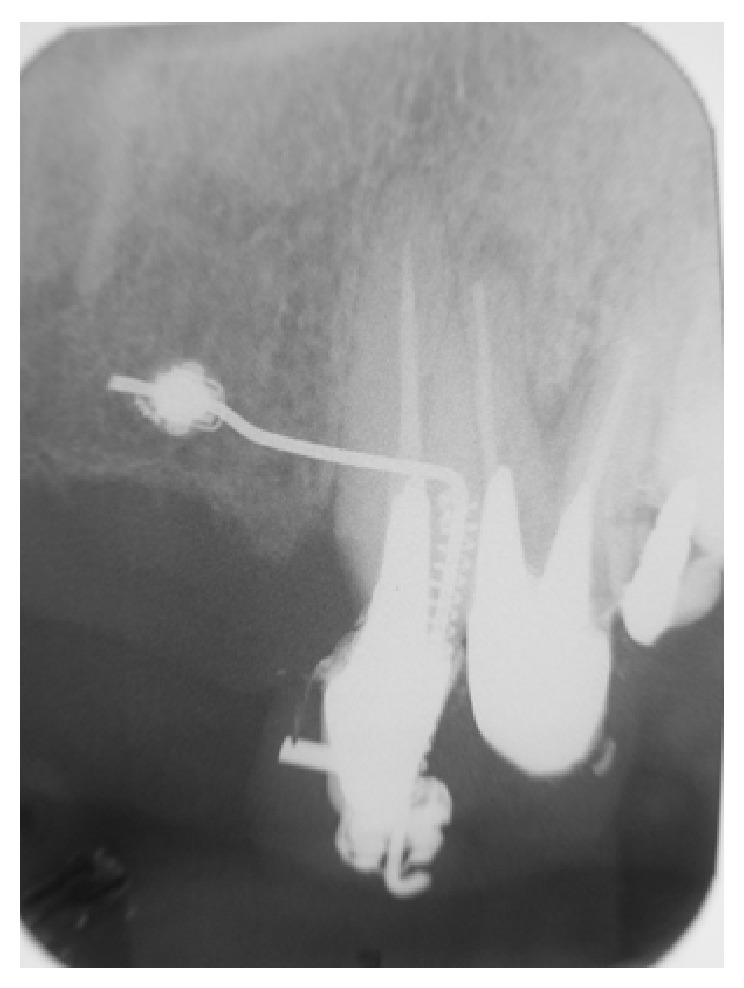
Radiographic illustration of extrusion appliance.

The postimplant clinical and radiographic status (Figures 7 and 8) showed good positioning of the miniscrew in relation to the cuspid.

**Figure 9:**
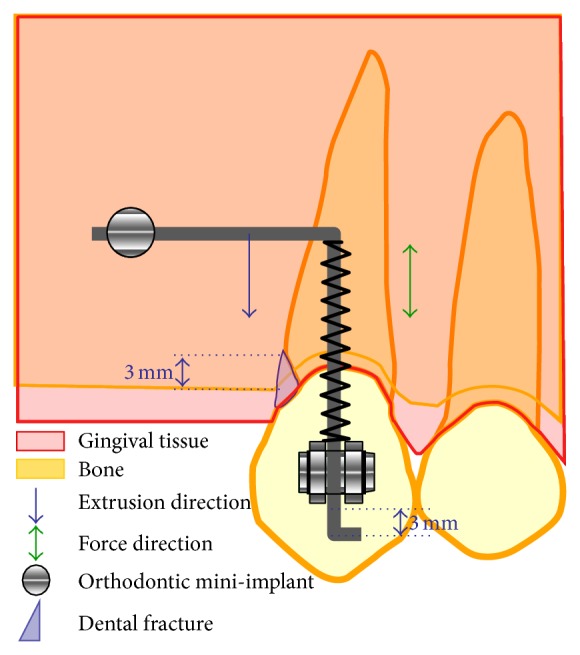
Illustration of extrusion appliance.

This appliance was adapted from the model described by Roth et al. [7], which replaces the horizontal bar. This model was utilized by the authors in the forced extrusion of two pillar teeth for regularization of the gingival margins with a single wire (vertical and horizontal segments) using a miniscrew as orthodontic anchorage. This mechanism transmitted a continuous force of 75 g onto the crown of tooth 23 to extrude its left upper cuspid (Figure 9).

Immediately after the installation of the miniscrew, a rectangular stainless steel wire (0.019′′  ×  0.025′′), folded perpendicularly at 90°, was connected to both the miniscrew (mesial part of the horizontal segment) and the vertical slot of the bracket (Morelli, Sorocaba, Brazil, as per Roth prescription) on the left upper cuspid (distal end of the vertical segment). A NiTi 0.25 × 0.76 mm open coil spring (Morelli, Sorocaba, Brazil), inserted in the vertical segment of the wire and welded to the top part of this segment, immediately transmitted a load force of 75 g onto the bracket, thus forcing the extrusion of the tooth up to the stop determined by a second 90° fold in the wire. This stop mechanism allowed the presetting of the exact extrusion amount (3.0 mm). The force of 75 g was checked with a dynamometer (Correx Swiss, Haag-Streit Bergen, 10 to 250 cN).

The implant did not impair the patient's oral hygiene or eating habits, and the esthetic disturbance was not severe. Two days after the placement of the appliance, the incisal and palatal edges were gradually shortened to provide sufficient space for the extrusion.

## 3. Results

**Figure 10:**
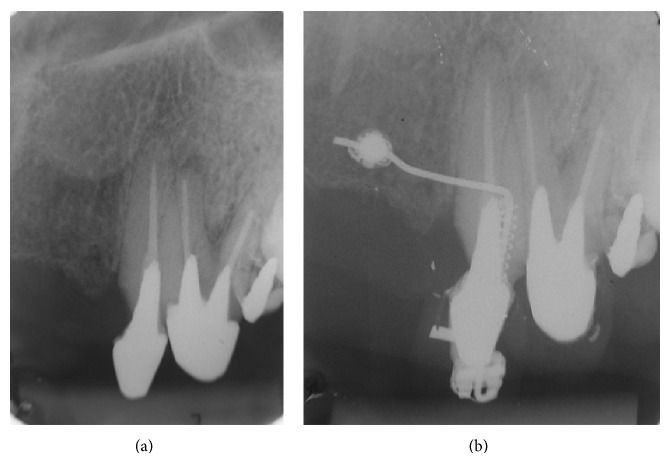
Initial and final radiographs.

**Figure 11:**
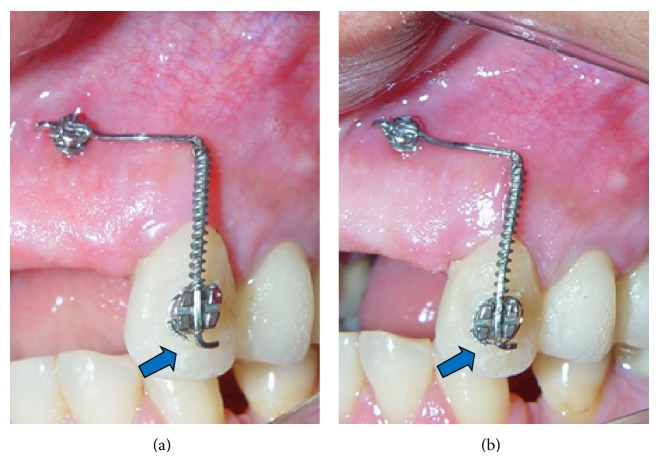
Initial and final clinical situation of extruded left upper cuspid (tooth 23). On the day of the patient's discharge, the tooth had normal periodontal probing depth (less than 3 mm all around).

After an 11-day period of forced extrusion, both clinical and radiographic analyses (Figures 10 and 11) indicated no problems with the miniscrew, such as peri-implant inflammation and root reabsorption.

**Figure 12:**
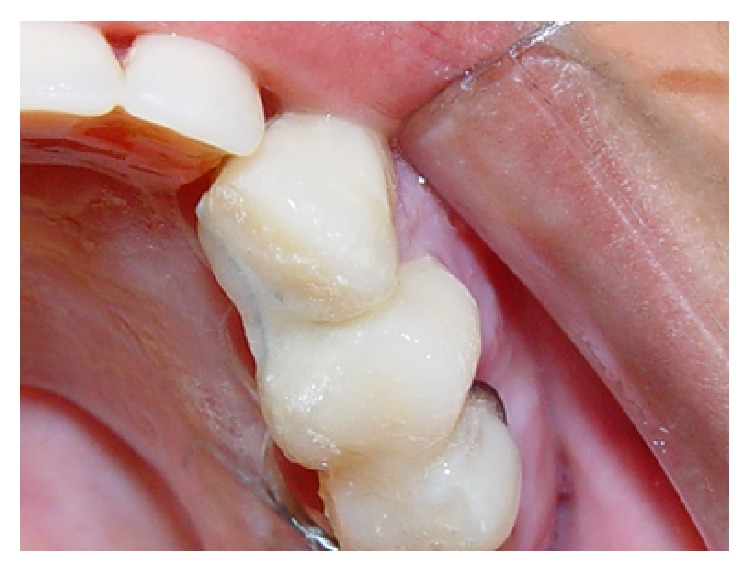
Fixed retainer of tooth 23. It must be used until the future prosthetic restoration can be installed.

After removing the implant, the palatine surfaces of the crowns of the left upper cuspid and left upper premolar were connected with a 0.019 × 0.025′′ stainless steel wire and acrylic resin (Figure 12) until the future prosthetic restoration was installed.

As at the initial planning, the supporting teeth of the old fixed prosthesis (24, 23, and 13–16) would be restored with unitary fixed prostheses and the absent anterior teeth (22, 21, 11, and 12) would be rehabilitated with prostheses on dental implants.

## 4. Discussion

Miniscrews are commonly used for temporary orthodontic anchorage and are usually removed relatively soon after treatment. There is no consensus in the literature about miniscrew osseointegration [8–10]. In this case report, the miniscrew stood in place for 11 days, and we believe that there was an overlap of the miniscrew with only trabecular and cortical bones.

Conventional implants are subject to high intermittent forces of mastication. By contrast, forces acting on orthodontic anchors are light and continuous. Miniscrew implants are attached mechanically to the bone with no intent to encourage or establish any form of osseointegration and are removed as soon as they have served their purpose [11]. In addition, several studies have suggested that the healing periods of these small temporary anchorage devices can be shortened, in contrast to large endosseous implants [12]. Because miniscrews were used for only short periods of time and there were only light and continuous forces acting on the orthodontic anchors, the appliance used in this study could be loaded immediately after its installation. As full osseointegration of screws used in orthodontic applications is a disadvantage that complicates the removal process, most of these devices are manufactured with a smooth surface, thereby minimizing the development of bone ingrowth [13].

The crown-lengthening surgery is performed to increase the clinical crown length without violating the biologic width [2]. Several techniques have been proposed for clinical crown-lengthening, including gingivectomy, apically displaced flap with or without resective osseous surgery, and surgical extrusion using a periotome [2]. Forced tooth eruption via orthodontic extrusion is the technique of choice when clinical crown-lengthening is necessary in the esthetic zone [14–16]. Some authors [2, 14, 15] affirm that, after clinical and radiographic evaluation, the surgical extrusion technique offers several advantages over the other conventional surgical techniques such as preservation of biologic width, interproximal papilla, and gingival margin position. Additionally, it maintains the esthetics, prevents marginal bone loss, and exposes sound tooth structure for the placement of restorative margins [14, 15].

We decided to extrude the tooth by 3 mm; this decision was based on the periapical radiography, which measured 2 mm from the edge of the fracture to the alveolar crest, to which we added 1 mm to restore the biological space, therefore totaling 3.0 mm. At the end of the extrusion, a suggestive periapical image of radiolucency was observed; however, the radiolucent image could correspond to the space derived from the tooth extrusion.

As at the initial planning, following an inspection of the length of the filling material of tooth 23, it was decided that the tooth would be retreated endodontically along with teeth 24, 25, 26, 27, 15, 16, 45, 44, and 35.

Furthermore, considering that the long span of the fixed prosthesis could have overloaded the canine, there could have been an inadequate tension in the cervical third of the intraradicular retainer due to inappropriate length. The design of the future prosthetic restoration would contain intraroot retainers and separate (unitary) fixed prostheses in teeth 23, 24, 13, 14, 15, and 16 as well as 45, 44, 35, 25, 26, and 27. The absent anterior teeth (22, 21, 11, and 12) would be rehabilitated with prostheses on dental implants.

Miniscrews for orthodontic treatments are available in several lengths (5–12 mm) and diameters (1.2–2.0 mm) [17]. E. Mizrahi and B. Mizrahi [11] recommended the use of miniscrews with a diameter of 1.5 mm because these implants are usually installed in the interdental root spaces. However, there should be caution when setting the anchorage devices to avoid any potential damage to nearby anatomical structures, such as roots or periodontal ligaments. This possible damage could result in an unintended mobility of the miniscrews and, consequently, in a failure of the implant [8, 18]. In the present case, the diameter of 2.0 mm was chosen to guarantee higher stability of the orthodontic anchor and because there were sufficient bone tissue and no dental roots at the site where the miniscrew was placed.

Although there is no consensus [19] about the miniscrew insertion procedure, it can be easily carried out in the practice setting by a clinician or an orthodontist and will take only a few because it requires only the direct transmucosal placement of the miniscrew [20]. The device proposed in this case report uses a self-drilling mini-implant that is inexpensive, is easily implemented, is predictable enough to be used routinely in practice, and is safer [20] than other techniques (e.g., miniplates).

We utilized the periapical radiograph (parallelism) technique as a presurgical guide for the correct placement of the self-drilling miniscrew. Normally, presurgical computerized tomography (CT) would be required if there were limited interradicular spaces between roots or anatomical details (e.g., danger of maxillary sinus perforation) around the target point [21] because it is necessary to ensure the safe placement of the miniscrew. CT was not needed in this specific case as we were dealing with an edentulous area [6], which was distant from the tooth roots.

Placement protocols varied markedly [9, 17]. One study have compared surgical techniques with and without drilling and found that self-drilling screws had significantly more bone-implant contacts and a higher stability. In the present case, we used self-drilling miniscrews installed by means of a hand driver and placed transmucosally to reduce patient discomfort [3].

Cho et al. [22] showed that counterclockwise rotational moments of 2 Ncm (obtained by applying a force of 284 g) can be a risk factor for miniscrew stability. In this case, the construction of the system generated a vertical force (75 g) that was not sufficient to rotate the miniscrew counterclockwise, and, additionally, the rotational movement was limited by the vertical slot of the bracket.

Miniscrews are useful devices for various orthodontic teeth movements because there are few anatomic limitations to their placement, their medical cost is low, and they can be installed with minimum surgical trauma [3]. The present case report demonstrated the successful use of a miniscrew as an anchoring device during a dental extrusion with no involvement of other teeth, implant side effects, or aesthetic impairment of the gingival margin.
